# The Effects of Prolonged Storage on ARPE-19 Cells Stored at Three Different Storage Temperatures

**DOI:** 10.3390/molecules25245809

**Published:** 2020-12-09

**Authors:** Rakibul Islam, Rima Maria Corraya, Lara Pasovic, Ayyad Zartasht Khan, Hans Christian D. Aass, Jon Roger Eidet, Tor Paaske Utheim

**Affiliations:** 1 Department of Medical Biochemistry, Oslo University Hospital, 0450 Oslo, Norway; rimamaria10@gmail.com (R.M.C.); larapasovic@gmail.com (L.P.); a.a.z.khan@studmed.uio.no (A.Z.K.); h.c.aass@medisin.uio.no (H.C.D.A.); j.r.eidet@gmail.com (J.R.E.); utheim2@gmail.com (T.P.U.); 2 Department of Surgery, Akershus University Hospital, 1478 Lørenskog, Norway; 3 Department of Ophthalmology, Oslo University Hospital, 0450 Oslo, Norway; 4 Department of Ophthalmology, Stavanger University Hospital, 4011 Stavanger, Norway; 5 Department of Ophthalmology, Sørlandet Hospital Arendal, 4838 Arendal, Norway

**Keywords:** retina, storage condition, temperature, regenerative medicine, cell therapy, age-related macular degeneration (AMD), oxidative stress

## Abstract

This study aimed to investigate how prolonged storage of adult retinal pigment epithelial (ARPE-19) cell sheets affects cell metabolism, morphology, viability, and phenotype. ARPE-19 cell sheets were stored at three temperatures (4 °C, 16 °C, and 37 °C) for three weeks. Metabolic status and morphology of the cells were monitored by sampling medium and examining cells by phase-contrast microscopy, respectively, throughout the storage period. Cell viability was analyzed by flow cytometry, and phenotype was determined by epifluorescence microscopy after the storage. Lactate production and glucose consumption increased heavily, while pH dropped considerably, through storage at 37 °C compared to 4 °C and 16 °C. During storage, morphology started to deteriorate first at 4 °C, then at 37 °C, and was maintained the longest at 16 °C. Viability of the cells after three weeks of storage was best preserved at 16 °C, while cells stored at 4 °C and 37 °C had reduced viability. Dedifferentiation indicated by reduced expression of retinal pigment epithelium-specific protein 65 (RPE65), zonula occludens protein 1 (ZO-1), and occludin after three weeks of storage was noticed in all experimental groups compared to control. We conclude that storage temperature affects the metabolic status of ARPE-19 cells and that 16 °C reduces metabolic activity while protecting viability and morphology.

## 1. Introduction

The retinal pigment epithelium (RPE) is a monolayer of pigmented cells located between the neurosensory retina and the choroid [1,2]. Loss and dysfunction of RPE cells lead to major pathological changes as seen in age-related macular degeneration (AMD), Stargardt disease, and other macular dystrophies [3]. Transplantation of tissue-engineered RPE cell sheets or suspensions offers the promise of a single-intervention cure [4,5,6,7]. A growing body of preclinical studies employing several animal models and various RPE cell sources supports the feasibility of this treatment [8,9,10,11,12,13,14,15,16,17,18,19,20,21,22]. Several clinical studies have demonstrated promising results [23,24,25,26]. Other studies assessing the transplantation of RPE cells derived from different sources are underway [27].

The cells can be delivered to the subretinal space by means of the cell suspension or as an RPE patch [28,29]. However, the preparation of RPE for transplantation in humans is a complex and costly procedure, and upcoming regulatory demands [30] are likely to lead to the establishment of specialized cell processing centers, as described by Oie et al. for human oral mucosal cells [31]. Development of a suitable storage method will be essential to enable the transportation of cell constructs from processing centers to clinics worldwide, thereby ensuring wider access to this novel treatment.

Through several studies [32,33,34,35,36], we and others have explored the feasibility of establishing a xenobiotic-free storage system for RPE cells above freezing temperatures. This would circumvent the need for cryoprotectants, which are known to cause damage to stored cells [37,38,39]. We showed that storage at 16 °C best preserves adult retinal pigment epithelial (ARPE-19) cells stored for one week compared to eight other temperatures [32]. In the present study, we investigated the effect of storage temperature on the metabolic shift of ARPE-19 cells and evaluated the consequences measured by morphology, viability and phenotypes.

## 2. Results

### 2.1. Effect of Three-Week Storage on the Metabolism of Cultured ARPE-19 Cells

**Figure 1:**
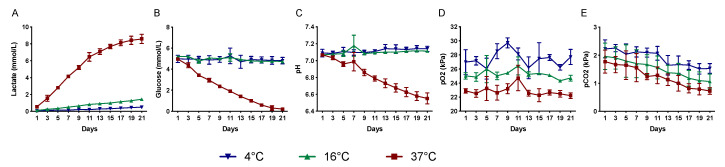
Effect of storage temperature on the metabolic function of RPE cells. The level of lactate (**A**), glucose (**B**), pH (**C**), pO_2_ (**D**), and pCO_2_ (**E**) in the storage medium was measured every alternate day during the storage period. Data are presented as mean ± standard deviation of the mean. (*n* = 8).

To study the effect of storage time and temperature on metabolic parameters (lactate, glucose, pH, pO_2_, and pCO_2_), the storage medium was sampled every alternate day. Lactate concentration was dramatically increased after storage at 37 °C (0.5–8.6 mmol/L), while it was only slightly increased after storage at 4 °C and 16 °C (0.1–0.5 mmol/L and 0.1–1.4 mmol/L, respectively) (Figure 1A).

The glucose concentration decreased markedly in cultures stored at 37 °C for three weeks (5.0–0.2 mmol/L), while only a slight reduction was noted in cultures stored at 4 °C and 16 °C (5.0–4.8 mmol/L and 5.2–4.7 mmol/L, respectively) (Figure 1B). The pH was maintained at 7.1 at 4 °C and 16 °C throughout the three weeks of storage but gradually declined at 37 °C storage from 7.1 to 6.6 (Figure 1C). The pO_2_ was maintained in all temperature groups throughout the storage period (27.0–27.7 kPa at 4 °C, 25.0–24.7 kPa at 16 °C, and 22.9–22.2 kPa at 37 °C) (Figure 1D). The pCO_2_ decreased gradually in all storage groups (2.2–1.5 kPa at 4 °C, 2.0–1.1 kPa at 16 °C, and 1.8–0.7 kPa at 37 °C) (Figure 1E). The partial pressure of both O_2_ and CO_2_ was inversely proportional to the storage temperature. The metabolic investigations thus show that during the storage duration, the 16 °C conditions kept the measured parameters more stable than the 4 °C and 37 °C groups.

### 2.2. Effect of Three-Week Storage on the Morphology of Cultured ARPE-19 Cells

**Figure 2:**
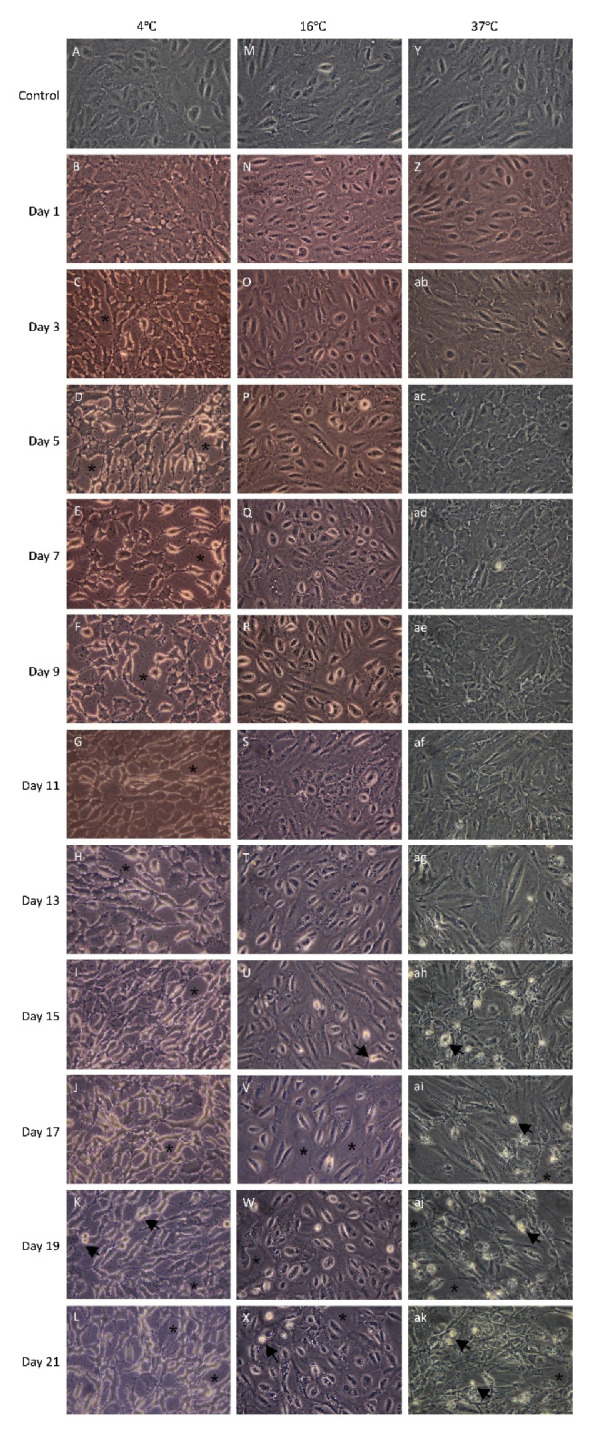
Effect of storage temperature on the morphology of adult retinal pigment epithelial (ARPE-19) cells. Phase-contrast photomicrographs were captured every alternate day during the storage period. Photomicrographs **A**, **M** and **Y** show ARPE-19 cell cultures before storage at three different temperatures. Photomicrograph **B**–**L**, **N**–**X**, and **Z**–**ak** demonstrate the morphology of the ARPE-19 cell cultures following 1 to 21 days of storage at 4 °C, 16 °C and 37 °C, respectively. Black arrows indicate apoptotic cells. Asterisks indicate intercellular spacing (magnification: 400×; *n* = 4).

To study the morphological effects of storage duration at the three different storage temperatures, phase contrast photomicrographs were captured every alternate day. Prior to storage, cells were generally well apposed and showed typical ARPE morphology (Figure 2A,M,Y).

At the end of the three-week storage period, cells stored at 16 °C showed a morphology most similar to the control. Signs of apoptosis (marked with a black arrow) and intercellular spacing (marked with an asterisk; Figure 2N–X) were infrequently observed at this storage temperature. The majority of cells stored at 4 °C and 37 °C showed signs of cell damage, apoptosis, and necrosis. These signs included extensive loss of cell–cell contact, detachment from the surrounding cells and shrinkage of cytoplasm. In the 4 °C storage group, the deformation of cells was evident from day one (Figure 2B) when the cells started to shrink. In the 37 °C group, cell detachment and fragmentation into apoptotic bodies were observed from day 13 (Figure 2ag–ak). The morphological evidence suggests that both 4 °C and 37 °C storage conditions are suboptimal for maintaining the morphology of the cells, while 16 °C preserved it for the longest duration.

### 2.3. Effect of Three-Week Storage on the Viability of Cultured ARPE-19 Cells

**Figure 3:**
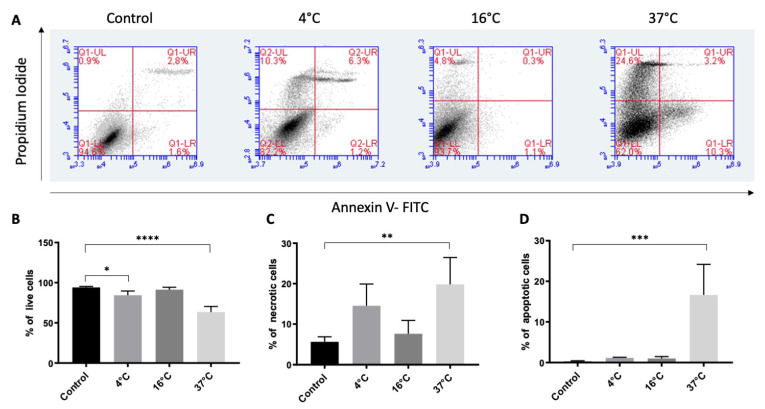
Effect of storage temperature on the viability of ARPE19 cells. Live, necrotic and apoptotic cells were detected by flow cytometry using annexin V and propidium iodide (PI). Cultured ARPE-19 cells were stored at three temperatures for three weeks. Dot plots (**A**) from the flow cytometry analysis were gated based on unstained cells for each experiment (not shown). The cell populations were distributed in four quadrants where the lower left quadrant represents live cells (annexin V and PI-negative), the upper left and right quadrants together represent necrotic cells (only PI-positive as well as both annexin V and PI-positive), while the lower right quadrant represents apoptotic cells (annexin V-positive and PI-negative). Control cells were not stored. The bar chart illustrates the percentages of live (**B**), necrotic (**C**), and apoptotic (**D**) cells. Data are presented as the mean ± standard deviation of the mean of four independent experiments. * *p* < 0.05, ** *p* < 0.01, *** *p* < 0.001, and **** *p* < 0.0001.

To assess cell survival after storage at 4 °C, 16 °C and 37 °C for three weeks, cell viability was analyzed by measuring annexin V-binding and PI uptake using flow cytometry (Figure 3A).

Cell viability after three weeks of storage (defined as the percentage of cells that were annexin V- and PI-negative) was significantly reduced at 4 °C (84% ± 5%, *p* = 0.047) and 37 °C (63% ± 6%; *p* < 0.001), but not at 16 °C (91% ± 2%; *p* = 0.84), compared to the control (94% ± 1%) (Figure 3B). Necrotic cells, which were annexin V-negative and PI-positive, were significantly increased at 37 °C (19% ± 6%; *p* < 0.001), but not at 4 °C (14% ± 5%, *p* = 0.07) or 16 °C (7% ± 3%, *p* = 0.92), compared to the control (5% ± 1%) (Figure 3C). Similarly, the percentage of annexin V-positive and PI-negative apoptotic cells was increased only at 37 °C (16% ± 7%; *p* < 0.001), but not at 4 °C (1% ± 0.2%; *p* = 0.99) or 16 °C (1% ± 0.5%; *p* = 0.99), compared to the control (0.2% ± 0.2%) (Figure 3D). The viability analysis thus indicates that among the three storage conditions tested here 16 °C condition is better for preserving cell viability similar to the non-stored cells.

### 2.4. Effect of Three-Week Storage on the Phenotype of Cultured ARPE-19 Cells

**Figure 4:**
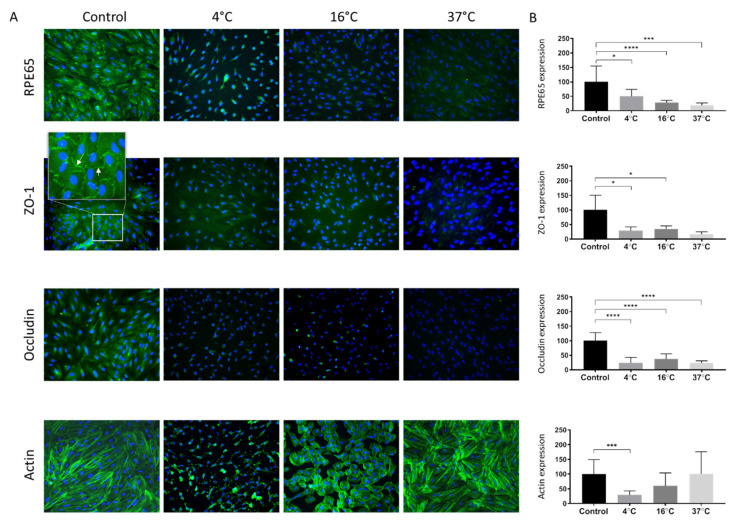
The effect of storage temperature on ARPE-19 cell phenotype. (**A**) The expression of RPE65, ZO-1 (white arrow points within a zoomed inset), occludin and actin in ARPE-19 cell cultures stored for three weeks at 4 °C, 16 °C, or 37 °C was compared with non-stored control cultures. Nuclear DNA was stained with 4′,6-diamidino-2-phenylindole (blue). (**B**) Expression of the markers quantified by measuring the total fluorescence intensity normalized by cell number. The bar charts show the fluorescence intensity of anti-RPE65, anti-ZO-1, anti-occludin, and anti-actin relative to control cultures (100%). Magnification 200×. Data are expressed as mean ± standard deviation of the mean. * *p* < 0.05, *** *p* < 0.001 and **** *p* < 0.0001

To study the effect of storage temperature on ARPE-19 phenotype following three weeks of storage at 4 °C, 16 °C, and 37 °C, the cells were immunostained with four different markers. The anti-RPE65 antibody was used to target an RPE-selective protein essential for the regeneration of visual pigment [40]. RPE65-expression normalized to control (set to 100%) appeared to be inversely proportional to the storage temperature (4 °C: 50% ± 24%, *p* = 0.011; 16 °C: 29% ± 7%, *p* < 0.001; 37 °C: 19% ± 8%, *p* < 0.001) (Figure 4A,B).

To assess the presence of intercellular tight junctions, staining with anti-ZO-1 and anti-occludin antibodies was performed. The ZO-1 marker localized to cell borders and was present between all apposed cells in the control group, indicating a tight junction organization typical of native RPE (Figure 4A). Compared to the non-stored control (set to 100%; Figure 4A,B) ZO-1-expression was reduced following storage at all storage temperatures (29% ± 13%, *p* = 0.035; 35% ± 10%, *p* = 0.0155 and 17% ± 8%, *p* = 0.0510 for 4, 16 °C and 37 °C, respectively). Occludin, another tight junction marker, was also significantly reduced following storage at all storage temperatures (24% ± 19%, *p* < 0.001; 37% ± 18%, *p* < 0.001; and 24% ± 7, *p* < 0.001; for 4 °C, 16 °C, and 37 °C, respectively) compared to the non-stored control (set to 100%; Figure 4A,B).

Alexa Fluor 568 phalloidin staining was applied for selective labeling of F-actin in order to visualize the cytoskeleton and evaluate the formation of stress fibers. Actin staining revealed a continuous cytoplasmic network of filamentous structures in the control cultures, with the formation of stress fibers seen in some cells (Figure 4A). After storage at 4 °C, there was disorganization and complete loss of actin filamentous structure (Figure 4A). However, in the 16 °C group, actin filaments were less stretched and more circular, whereas the filaments were maintained after storage at 37 °C compared to the control (Figure 4A). Measuring the fluorescence intensity of the filament staining showed that at 4 °C storage it was significantly lower (28% ± 14%; *p* < 0.001) compared to the control (Figure 4B), while, there was no statistically significant difference at 16 °C storage (60% ± 43%; *p* = 0.070) at 37 °C (100% ± 75%; *p* > 0.999) after three weeks (Figure 4A,B).

## 3. Discussion

In this study, we investigated how prolonged storage of ARPE-19 cell sheets affects cell metabolism, morphology, viability, and phenotype. We found that the temperature affects the metabolic shift over time. Among the three temperature groups, 16 °C kept the metabolic shift low, cell viability high, and morphology preserved. However, the phenotype was not maintained at control levels after storage at any of the temperatures.

Our results demonstrated an increased breakdown of glucose to lactate with a concomitant reduction in pH during storage at 37 °C compared to 4 °C and 16 °C. This is in accordance with earlier findings in stored human-induced pluripotent stem cell-derived retinal pigment epithelium cells [36], epidermal cell sheets [41], cultured human conjunctival cells [42] and human oral keratinocytes [43]. The high lactate/glucose ratio indicates that the glycolytic pathway accounts for a large part of energy production from glucose and could possibly represent a cellular adjustment to avoid an excessive production of damaging reactive oxygen species generated through the oxidative phosphorylation pathway [44]. Lactate concentration at 37 °C storage rose linearly until day 11, after which it started to level off, possibly due to accelerated cell death. In fact, when evaluating the corresponding microscopy images of the same storage group, it appears that apoptotic bodies started to form after day 11. This could be related to the considerable drop in pH at 37 °C storage, which can induce cell apoptosis [45]. Since the storage media is easily accessible without affecting the cells, therefore, the lactate, glucose, and pH values together may be considered as critical quality control parameters for RPE-cells during storage at 37 °C. At 4 °C and 16 °C storage, the changes in the metabolic parameters were not as dramatic as 37 °C. At 4 °C storage, cells exhibited typical signs of apoptosis and necrosis early on during storage without any obvious connection to metabolic parameters. At 4 °C, cells die mainly because of low temperature-related stress [32], whereas, at 37 °C, the primary causes may be associated with accumulation of lactate, pH reduction and associated apoptosis [43,45]. At 16 °C, the morphology of the cells was maintained the longest without dramatic changes in metabolic parameters. Thus, storage at 16 °C reduced the metabolic rate of the cells while not exerting the detrimental effect of low temperature-associated stress [43].

This explanation is corroborated by the post-storage viability, which demonstrated that after 16 °C storage, the live-cell percentage did not significantly differ from non-stored control cells (Figure 3). In a study by Kitahata et al., cell suspension of human-induced pluripotent stem-cell-derived retinal pigment epithelium cells demonstrated a higher percentage of viable cells at 16 °C compared to 4 °C, 25 °C and 37 °C following 24 h of storage [36]. However, our previous study demonstrated the viability of about 50% after one-week storage at 16 °C [32]. This discrepancy can be explained by a change in the viability assessment analysis. In the present study, we used flow cytometry to measure the expression of the apoptotic marker, phosphatidylserine, by binding of annexin V and determine the dead cells by PI staining. Viability was calculated as the percentage of non-stained cells from total acquired cells suspension for the analysis. In the previous study, viability was not assessed in the suspended cells, rather as fluorescence intensity of calcein-acetoxymethyl ester staining on adherent cells determined by a microplate fluorometer.

The RPE is a highly specialized tissue performing several functions that are crucial for sight, including phagocytosis of shed photoreceptor outer segments, regeneration of visual cycle pigments, and transport of nutrients and fluid between the choroid and neuroretina [3,46]. These traits are affected by macular disease and could be remedied by transplanted tissue. It is, therefore, important that transplanted cells display differentiated RPE properties. The ARPE-19 cell line is a widely employed model for the study of RPE biology. While it displays significant functional differentiation [47,48], it does not mirror all characteristics of native RPE, and its phenotype is highly dependent on culture conditions [49,50,51,52]. However, the use of serum-free media and plastic substrates, which are employed herein, have been shown to reduce dedifferentiation in culture [49,53]. Earlier, we showed that ARPE-19 cells stored at 16 °C for one week are capable of maintaining the expression of the RPE differentiation marker RPE65 [32]. The current results demonstrated a reduced RPE65 expression at 16 °C following three weeks’ storage compared to one-week storage, which may indicate dedifferentiation of ARPE-19 cells with increasing storage duration. Similarly, we earlier showed maintained expression of the tight junction markers ZO-1 and occludin after one-week storage of ARPE-19 cells [32,33]. The expression of these markers is not maintained after three weeks’ storage. The effect of storage on cell phenotype has been described previously for several cell types. Studies have demonstrated that cultured limbal cells can be stored for one week in an organ culture medium at 23 °C with intact phenotype [54]. Similarly, cultured human oral keratinocytes can be stored under the conditions described herein for one week without signs of differentiation [43]. Microarray analysis demonstrated upregulation of tight junction proteins after one-week storage at 37 °C compared to 12 °C, indicating an increased synthesis of tight junctions in HOK cells stored at 37 °C [55]. In cultured epidermal cell sheets stored at different temperatures for two weeks, there was a tendency of increased expression of differentiation markers at all temperatures except for 12 °C [56]. Based on these observations, it seems that the phenotypic plasticity during storage varies between different cell types.

In the present study, there were also changes in the distribution of the actin cytoskeleton, which is important for cell adhesion, morphogenesis, and phagocytosis. Contractile actomyosin bundles called stress fibers assemble following mechanical stress and are common in cultured epithelial cells [57,58]. Actin staining revealed a continuous cytoplasmic network of filamentous structures in the control cultures, with the formation of stress fibers seen in some cells. These features were maintained after storage at 37 °C. After storage at 4 °C and 16 °C, however, there was a disruption of the actinic cytoskeleton. Disrupted staining patterns of the actin cytoskeleton, tight junctions, and adherens junctions in the RPE in relation to elevated reactive oxygen species were previously reported elsewhere. [59] ARPE-19 cells stored at 16 °C for one week displayed a similar distribution with a predominantly circumferential actin arrangement and fewer elongated cells than control cultures [32].

Replacement of the diseased RPE is on the verge of becoming a reality in regenerative therapies to cure age-related macular degenerative diseases. The successful outcome with the first two patients from a clinical study by transplanting cultured cell sheet has demonstrated the potential effectiveness of the therapy [26]. The development of complementary storage techniques for RPE transplants is likely to have a large medical impact as it allows flexibility in scheduling surgery and can widen patients’ access to future applications of regenerative therapy.

We conclude from our study that the storage temperature affects the metabolic status of ARPE-19 cells and that 16 °C is superior for keeping the metabolic activity low while protecting the viability and morphology. Our study infers the importance of monitoring metabolic parameters as quality control of the stored ARPE-19 cells.

## 4. Materials and Methods

### 4.1. Cell Culture Media and Reagents

ARPE-19 cells were obtained from the American Type Culture Collection (ATCC) (Manassas, VA, USA). Dulbecco’s Modified Eagle’s Medium: nutrient mixture F12 (hereafter named DMEM:F12), fetal bovine serum (FBS), bovine serum albumin (BSA), trypsin-EDTA, 4-(2-hydroxyethyl)-1-piperazineethanesulfonic acid (HEPES), sodium bicarbonate, gentamycin, phosphate-buffered saline (PBS), penicillin, streptomycin, 4′,6-diamidino-2-phenylindole (DAPI), propidium iodide (PI), Tween-20 and PAP pen were purchased from Sigma-Aldrich (St. Louis, MO, USA). Fluorescein isothiocyanate (FITC)-labeled annexin V (to bind PS), annexin V-binding buffer containing 10 mM HEPES (pH 7.4), 140 mM NaCl, and 2.5 mM CaCl_2_, were purchased from Becton Dickinson Biosciences (BD), Belgium. Minimum essential medium (MEM) was purchased from Invitrogen (Carlsbad, CA, USA). Pipettes, 25 cm^2^ flasks, 15 mL and 50 mL centrifugation tubes, 1 L glass bottles, and pipette tips were supplied by VWR International (West Chester, PA, USA). Vacuum filtration rapid filter mix was supplied by BioNordika (Oslo, Norway). Mouse anti-RPE65, rabbit anti-occludin, FITC-conjugated goat anti-mouse IgG and FITC-conjugated goat anti-rabbit IgG antibodies were obtained from Abcam (Cambridge, UK). Mouse anti-ZO-1 and Alexa Fluor 568 phalloidin were purchased from Life Technologies (Carlsbad, CA, USA).

### 4.2. Culture of ARPE-19 Cells

Human ARPE-19 cells were routinely cultured in 95% air and 5% CO_2_ at 37 °C in DMEM:F12 containing 10% FBS, 50 units/mL penicillin and 50 µg/mL streptomycin. The cells at passage 6 were seeded (120,000 cells/flask) in 25 cm^2^ culture flasks with filter closer. The culture medium was changed every other day, and confluent cultures were obtained on the sixth day. Control cultures, which were not subjected to subsequent storage, were immediately prepared for the various analyses.

### 4.3. Storage of ARPE-19 Cells

After the six-days culture period, the T25 flasks were removed from the incubator, and the culture medium was replaced by a storage medium consisting of 9.53 g MEM, 25 mM HEPES, 600 mg/L sodium bicarbonate and 50 µg/mL gentamycin in 1 L distilled water. The filter cap of the flasks was changed to a solid cap to avoid evaporation during storage. Thereafter, the cultures were randomized for storage at three temperatures (4 °C, 16 °C and 37 °C) for three weeks in storage containers without CO_2_ supply. The storage containers have been described previously [32]. The stability of the temperature inside the storage containers has been reported [60] and was controlled regularly throughout all experiments.

### 4.4. Metabolic Analysis

Samples of the storage medium (2 mL) were taken every alternate day from day 1 to day 21 and were analyzed using a Radiometer ABL 700 blood gas analyzer (Radiometer, Bronshoj, Denmark) at room temperature. The following parameters were studied: pH, glucose, lactate, partial pressure of oxygen (pO_2_) and partial pressure of carbon dioxide (pCO_2_). The experiment was repeated eight times (*n* = 8).

### 4.5. Morphology Analysis

Morphology of the stored ARPE-19 cell cultures was assessed by light microscopy every alternate day during the storage period. The experiment was repeated four times (*n* = 4). Photomicrographs were captured at 400× magnification using a Leica DM IL LED microscope and a Canon EOS 5D Mark II camera.

### 4.6. Viability Analysis

Viability after three weeks of storage was analyzed by a flow cytometer (BD Accuri C6 flow cytometer, Becton Dickinson, CA, USA) using FITC-conjugated annexin V and PI. Annexin V binds selectively to phosphatidylserine (in the presence of calcium ions), which is anchored at the cytosolic face of the plasma membrane in viable cells. During the early phases of apoptosis, phosphatidylserine is re-localized to the outer surface of the plasma membrane, where it can be detected with fluorescently labeled annexin V [61]. Apoptotic cells were defined as annexin V-positive and PI-negative. PI passes through permeable cell membranes of necrotic cells and stains double-stranded DNA. Viable cells were defined as both annexin V- and PI-negative.

The analysis was performed according to the protocol provided by the supplier and repeated four times (*n* = 4). Briefly, RPE cells were trypsinized and centrifuged at room temperature. The supernatant was aspirated from the cell pellet, which was resuspended in 200 µL of annexin V-binding buffer containing annexin V-FITC (1 µL/mL) and incubated for 25 min at room temperature. PI dye (10 µg/mL) was added for further 5-min incubation at room temperature before the suspension was analyzed using a flow cytometer.

### 4.7. Phenotype Analysis

Cells were cultured in T25 flasks and stored at 4 °C, 16 °C and 37 °C for three weeks, as described above. Samples were subsequently prepared for immunocytochemical characterization with 30 min of 4% formaldehyde fixation at room temperature followed by one hour of permeabilization and blocking in PBS containing 1% BSA and 0.01% Tween-20. Control cells were processed for immunocytochemistry immediately after the six-day culture period. Anti-RPE65 (1:50), anti-ZO-1 (1:50) and anti-occludin (1:50) antibodies were diluted in blocking solution (PBS with 1% BSA). In the negative controls, primary antibodies were substituted with PBS. Samples were incubated for one hour at room temperature. FITC-conjugated goat anti-mouse secondary antibodies (diluted 1:200 in blocking solution) and FITC-conjugated goat anti-rabbit secondary antibodies (1:250) were added for one hour at room temperature. Specimens were washed three times in PBS, and 1 µg/mL DAPI was added during the last wash to stain the nuclear DNA. To visualize the actin cytoskeleton, samples were fixed, blocked, and permeabilized as described above and stained with 100 units/mL, which is equivalent to approximately 20 µM Alexa Fluor 568 phalloidin. After incubating for 1 h at room temperature, specimens were washed in PBS and stained with DAPI.

The samples were studied using a Nikon Eclipse Ti fluorescence microscope and photographed at ×200 magnification with a DS-Qi1 black-and-white camera. Identical exposure length and gain were maintained for all compared samples, and the image brightness was within the dynamic range of the camera. The experiments were repeated four times (*n* = 4).

The photomicrographs were then objectively assessed using ImageJ software (National Institutes of Health, Bethesda, MD, USA) as described previously [62], with some modifications. In brief, for DAPI count, 16-bit photomicrographs of DAPI-stained nuclei were converted to 8-bit images before being auto-thresholded to binary photos using the “Make Binary” function in ImageJ. Touching cell nuclei were separated by the “Watershed” command. Cell debris and other smaller cellular particles were excluded from analysis on the basis of size by the “Analyze Particle” function. For phenotypic quantification, unevenly transmitted light was subtracted from all 16-bit photomicrographs using the “Subtract Background (rolling = 50)” command in ImageJ before the total fluorescence intensity was measured. Finally, the total fluorescence intensity in each photomicrograph was divided by the number of DAPI-stained nuclei in each corresponding DAPI-photomicrograph. By using this method, we were able to normalize for differences in cell density in each photomicrograph.

### 4.8. Statistical Analysis

A one-way analysis of variance with Tukey’s post hoc comparisons (SPSS ver. 19.0 or GraphPad prism 8.2.1) was used for statistical evaluation of the results. *P* values below 0.05 were considered significant.
